# Artificial intelligence in positioning between mandibular third molar and inferior alveolar nerve on panoramic radiography

**DOI:** 10.1038/s41598-022-06483-2

**Published:** 2022-02-14

**Authors:** Eunhye Choi, Soohong Lee, Eunjae Jeong, Seokwon Shin, Hyunwoo Park, Sekyoung Youm, Youngdoo Son, KangMi Pang

**Affiliations:** 1 Department of Oral Medicine and Oral Diagnosis, School of Dentistry, Seoul National University, 101, Daehak-ro, Jongno-gu, Seoul, 03080 Republic of Korea; 2 Department of Industrial and Systems Engineering, Dongguk University - Seoul, 30 Pildong-ro 1-gil, Jung-gu, Seoul, 04620 Republic of Korea; 3 Department of Oral and Maxillofacial Surgery, Seoul National University Dental Hospital, 101 Daehak-ro, Jongno-gu, Seoul, 03080 Republic of Korea

**Keywords:** Anatomy, Medical research

## Abstract

Determining the exact positional relationship between mandibular third molar (M3) and inferior alveolar nerve (IAN) is important for surgical extractions. Panoramic radiography is the most common dental imaging test. The purposes of this study were to develop an artificial intelligence (AI) model to determine two positional relationships (true contact and bucco-lingual position) between M3 and IAN when they were overlapped in panoramic radiographs and compare its performance with that of oral and maxillofacial surgery (OMFS) specialists. A total of 571 panoramic images of M3 from 394 patients was used for this study. Among the images, 202 were classified as true contact, 246 as intimate, 61 as IAN buccal position, and 62 as IAN lingual position. A deep convolutional neural network model with ResNet-50 architecture was trained for each task. We randomly split the dataset into 75% for training and validation and 25% for testing. Model performance was superior in bucco-lingual position determination (accuracy 0.76, precision 0.83, recall 0.67, and F1 score 0.73) to true contact position determination (accuracy 0.63, precision 0.62, recall 0.63, and F1 score 0.61). AI exhibited much higher accuracy in both position determinations compared to OMFS specialists. In determining true contact position, OMFS specialists demonstrated an accuracy of 52.68% to 69.64%, while the AI showed an accuracy of 72.32%. In determining bucco-lingual position, OMFS specialists showed an accuracy of 32.26% to 48.39%, and the AI showed an accuracy of 80.65%. Moreover, Cohen’s kappa exhibited a substantial level of agreement for the AI (0.61) and poor agreements for OMFS specialists in bucco-lingual position determination. Determining the position relationship between M3 and IAN is possible using AI, especially in bucco-lingual positioning. The model could be used to support clinicians in the decision-making process for M3 treatment.

## Introduction

Mandibular third molar (M3) extraction is one of the most frequently performed surgical procedures in oral and maxillofacial surgery (OMFS). Among the complications following surgery, damage to the inferior alveolar nerve (IAN) is one of the most distressing, causing temporary or permanent neurosensory impairments in the lower lip and chin area at an incidence of 0.4% to 13.4%^1,2^. To avoid IAN damage, preoperative assessment of the position of the IAN in relation to the tooth is necessary. Panoramic radiography is used commonly to assess the relationship between M3 and IAN. Certain radiographic features such as darkening of the root and narrowing of the mandibular canal have been reported as risk factors for IAN injuries, although its clinical correlation was low^3^. Due to the development of cone-beam computerized tomography (CBCT), determination of positioning between the IAN and teeth has become more accurate, and CBCT is recommended before M3 extraction when the two aforementioned structures are superimposed on panoramic radiography^4^. However, the disadvantages of CBCT include higher radiation doses compared to two-dimensional imaging and the presence of image artifacts mainly produced by metal restorations^5^.

Therefore, accurate methods diagnosing the relationship between M3 and IAN on panoramic radiography are necessary. After the diagnostic methods determine whether both structures are truly in contact or intimate, assessment whether M3 is positioned lingually or buccally to the IAN is necessary to determine the direction of insertion of the surgical instruments.

Artificial intelligence (AI) models have reported excellent performance, mimicking the precision and accuracy of trained specialists in dentistry^6^. Various studies have applied AI algorithms to read panoramic radiographs for clinical conditions such as age estimation^7^, osteoporosis^8,9^, vertical root fracture^10^, automatic teeth detection and numbering^11^, apical lesions^12^, maxillary sinusitis^13^, detecting and segmenting the approximation of the inferior alveolar nerve and mandibular third molar^14^, periodontal bone loss^15^, gender determination^16^, and temporomandibular joint osteoarthritis^17,18^. Although some studies evaluate the relationship between M3 and IAN, those studies usually determine whether AI could determine M3 and IAN on panoramic radiograph or CBCT, which was easily discernible in human eyes^4,14,19,20^. A recent study predicting the difficulty of extraction by deep learning was easily distinguishable by humans^21^. In this study, we focused on the cases with M3 and IAN overlapping on panoramic radiograph and evaluated whether AI could determine if two structures were in contact or not and whether these structures were positioned buccally or lingually, which was difficult for humans to distinguish.

This study aimed to investigate the clinical use of an AI model developed to determinate the positional relationship between M3 and IAN from panoramic radiography using deep learning that compared the AI readings with those of OMFS specialists.

## Methods

### Materials

The written documentation of informed consent was waived and approved by the decision of the Institutional Review Board of Seoul National University Dental Hospital (ERI21004) and ethics committee approval for the study in the same institute was also obtained. All methods were performed in accordance with the relevant guidelines and regulation. Subjects were included retrospectively from an image database of patients who visited the Department of Oral and Maxillofacial Surgery at Seoul National University Gwanak Dental Hospital between January 2019 and December 2020. Patients who underwent both panoramic radiography (Kodak 8000 Digital Panoramic System, Trophy Radiologies, Carestream Health Inc., NY, USA) and CBCT (CS 9300, Carestream Health Inc., NY, USA) for M3 extraction with superimposition of M3 and IAN on the panoramic radiographs were selected. The patients consisted of 200 males and 194 females, with an age range of 20 to 72 years (mean ± SD age, 31.5 4 ± 9.96 years; range, 20 to 72 years). The panoramic images of 571 M3s from these patients were used in this study.

### AI model developments

The AI model was developed to evaluate two positional relationships between M3 and IAN.

1. Experiment 1: Determination of the true contact position between M3 and IAN.
2. Experiment 2: Determination of the bucco-lingual position between M3 and IAN.

**Figure 1:**
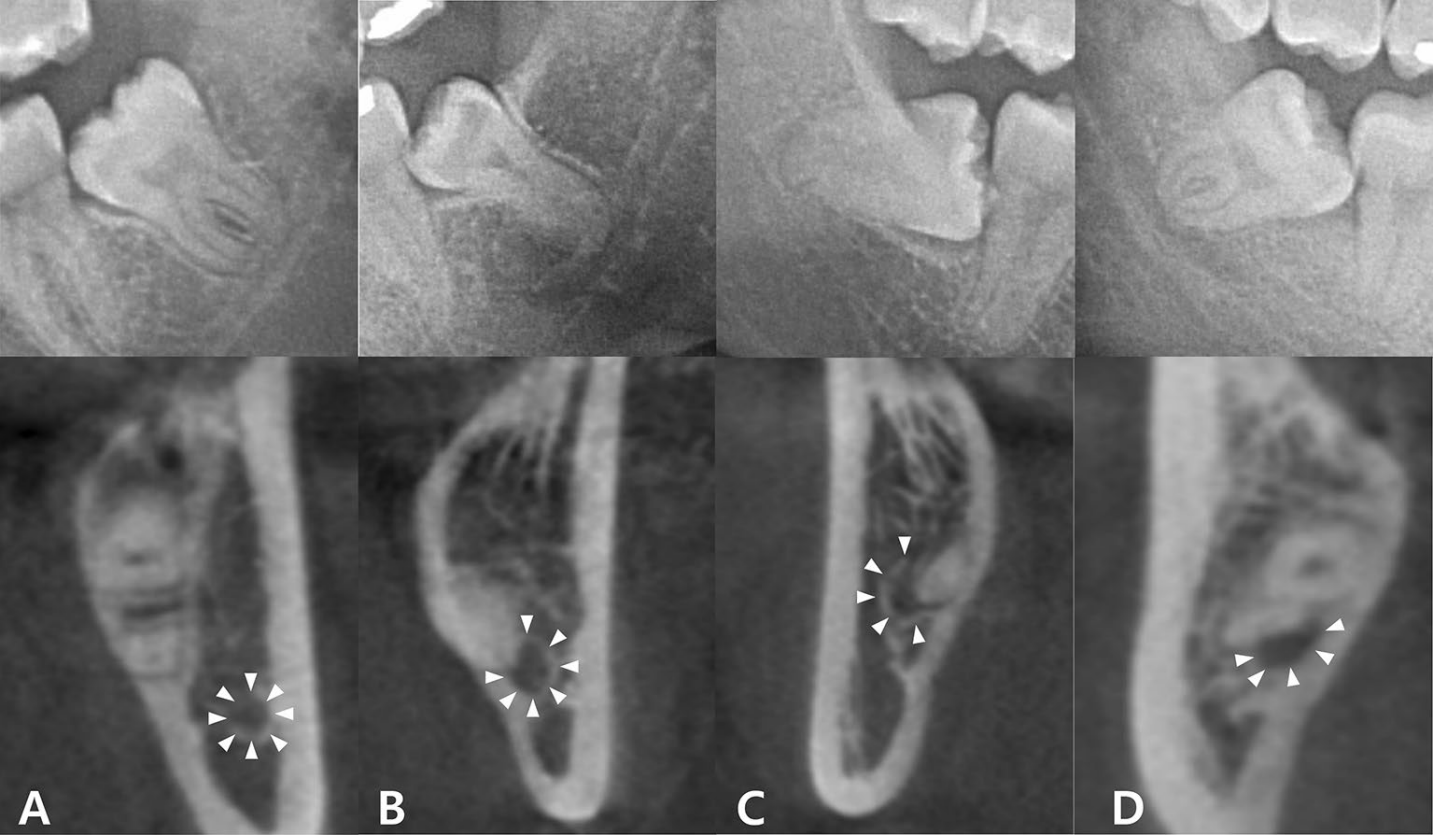
Classification of panoramic images based on CBCT. The M3 and IAN seemed to be superimposed in four panoramic images. White triangles point to the border of the IAN in CBCT. (**A**) Intimate but non-contact positioning between M3 and IAN. (**B**) True contact positioning between M3 and IAN. (**C**) IAN positioned buccal to M3. (**D**) IAN positioned lingual to M3. *CBCT* cone-beam computerized tomography, *M3* mandibular third molar, *IAN* inferior alveolar nerve.

Panoramic images that appeared overlapped were classified as true contact and intimate according to the presence or absence of the cortical line of the IAN canal on CBCT (Fig. 1A,B). Independently, the bucco-lingual positional relationship was also confirmed by CBCT (Fig. 1C,D). Determination of the positional relationship based on the CBCT was performed by an OMFR specialist (K.M. Pang). Among the 571 images, 202 were classified as true contact, 246 as false contact, 61 as IAN buccal position, and 62 as IAN lingual position. Regions of Interest (ROI) were extracted from the panoramic radiograph manually in JPG format with a matrix size of 400 × 400 pixels.

ResNet-50, mainly used for medical image classification^22^, is a substantially deeper and easier model to train compared to simple models such as VGGnet, and the core structure is a residual block^23^. Residual learning does not allow for error accumulation on the convolution layers but enables a better representation of the content in the convolution layers. By adopting a shortcut structure, the vanishing gradient issue is resolved^24^. Every image was resized to 224 × 224 pixels, and we randomly split the dataset into 75% for training and validation and 25% for testing. The model performance varies depending on the difficulty of data classification, so we performed 5 repeated experiments through random sampling. As a technical and strategic method to avoid overfitting, data augmentation was performed by image rotation ± 30 degrees, horizontal flipping, and brightness 20–80% for every mini-batch in training to compensate for the small number of data points to increase model robustness. In Experiment 1, a model was trained for 60 epochs with augmented data. The learning rate of the model was set to 1.0 × 10^–4^ and an Adam optimizer was used. In Experiment 2, training was progressed in 30 epochs with augmented data. In addition, the learning rate of the model was 1.0 × 10^–4^, and an Adam optimizer was used.

### Specialist performance analysis

To compare the accuracy between specialist and AI model, the same test dataset with the highest accuracy among the random samplings was selected for comparison with specialists in each experiment. Six oral and maxillofacial specialists with a mean 15.3 years (range from 8 to 30 years) of experience of third molar extraction were asked to assess the dataset. The specialists used a computer monitor for daily practice in their clinics, and the size of pixels was the same with images for AI, 224 × 224. For experiment 1, 100 images were analyzed; experiment 2 comprised 31 images. They had only one opportunity to analyze the images.

### Model and statistical analysis

Accuracy, precision, recall, F1 score, and AUC were calculated to evaluate each model performance. Accuracy is defined as the ratio of correct predictions. Precision is the ratio of true positives to true positives and false positives. Recall is the ratio of true positives to true positives and false negatives. F1 score is a harmonic mean of precision and recall: (2 × precision × recall)/(precision + recall), and AUC is the area under the ROC curve. The confidence intervals of AUC were found by bootstrapping with 1000 test sets sampled with replacement. For evaluation of AI clinical usability, the results between OPG reads by the AI and six OMFS specialists were compared. Accuracy, sensitivity, and specificity were calculated for diagnostic performance, and Cohen’s kappa was calculated to estimate the strength of agreement. Python programming language (v. 3.8.5), Tensorflow (v. 2.5.0), and a graphics card (GeForce RTX 3090) were used for analysis.

## Results

Table 1 shows the model performance in each experiment. The average accuracy, precision, recall, and F1 score were 0.63, 0.62, 0.63, and 0.61, respectively, in Experiment 1, with true contact position determination between M3 and IAN. The average accuracy, precision, recall, and F1 score were 0.76, 0.83, 0.67, and 0.73, respectively, in Experiment 2, with bucco-lingual position determination between M3 and IAN. The overall model performance was superior in Experiment 2 compared to Experiment 1.

**Table 1:** Model performance of five random samplings in each experiment.

Work	Accuracy	Precision	Recall	F1 score	AUC
**Experiment 1, true contact position between M3 and IAN**					
1	0.72	0.72	0.55	0.63	0.75
2	0.55	0.54	0.74	0.62	0.59
3	0.67	0.65	0.59	0.62	0.67
4	0.61	0.55	0.68	0.61	0.66
5	0.60	0.62	0.57	0.60	0.64
Average	0.63	0.62	0.63	0.61	0.66
**Experiment 2, bucco-lingual position between M3 and IAN**					
1	0.77	0.82	0.78	0.80	0.88
2	0.81	0.86	0.75	0.80	0.91
3	0.74	0.67	0.77	0.71	0.75
4	0.77	0.89	0.57	0.70	0.79
5	0.68	0.89	0.47	0.62	0.80
Average	0.76	0.83	0.67	0.73	0.83

*M3* mandibular third molar, *IAN* inferior alveolar nerve, *AUC* Area under the ROC curve.

**Figure 2:**
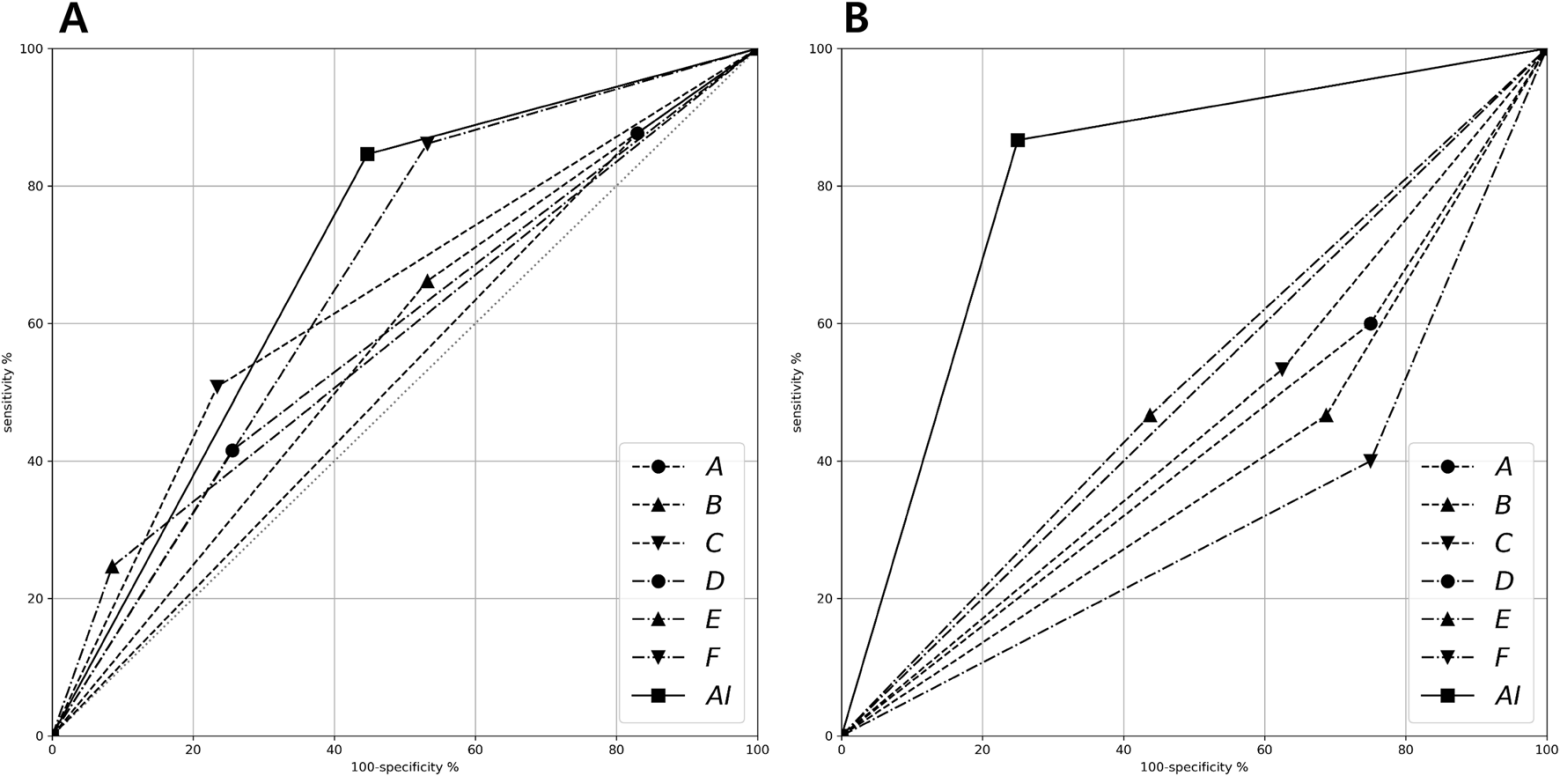
Comparison of sensitivities and specificities of six OMFS specialists and the AI model for determination of the positional relationship between M3 and IAN. (**A**) Experiment 1: Determination of true contact positioning between M3 and IAN. (**B**) Experiment 2: Determination of bucco-lingual positioning between M3 and IAN. *OMFS* oral and maxillofacial surgery, *AI* artificial intelligence, *M3* mandibular third molar, *IAN* inferior alveolar nerve.

The comparison of sensitivities and specificities between AI and OMFS specialists in each experiment is shown in Fig. 2. AI exhibited 72.32% accuracy in Experiment 1 and 80.65% in Experiment 2, but the highest accuracy among OMFR specialists in each experiment was 69.64% and 51.61%, respectively (Table 2). Cohen’s kappa of AI was highest in Experiment 2 and showed a substantial level of agreement (0.61), but those of OMFS specialists exhibited a slight to fair level of agreement. In both experiments, the AI read panoramic images more accurately than OMFS specialists, demonstrating higher diagnostic performance.

**Table 2:** Comparison of diagnostic performance across experiments.

Reader	Accuracy (%)	Sensitivity (%)	Specificity (%)	Cohen’s kappa	Kappa index
**Experiment 1, true contact position between M3 and IAN**					
A	58.04	87.69	17.02	0.05	Slight
B	58.04	66.15	46.81	0.13	Slight
C	61.61	50.77	76.60	0.26	Fair
D	55.36	41.54	74.47	0.15	Slight
E	52.68	24.62	91.49	0.12	Slight
F	69.64	86.15	46.81	0.35	Fair
AI	72.32	84.62	55.32	0.41	Moderate
**Experiment 2, bucco-lingual position between M3 and IAN**					
A	41.94	60.00	25.00	− 0.15	Poor
B	38.71	46.67	31.25	− 0.22	Poor
C	45.16	53.33	37.50	− 0.09	Poor
D	48.39	100.00	0.00	incalculable	incalculable
E	51.61	46.67	56.25	0.03	Slight
F	32.26	40.00	25.00	− 0.35	Poor
AI	80.65	86.67	75.00	0.61	Substantial

*M3* mandibular third molar, *IAN* inferior alveolar nerve.

## Discussion

This study evaluated if AI could determine the positional relationship between M3 and IAN based on panoramic radiography regarding whether the two structures were in contact or intimate and whether the IAN was positioned lingually or buccally to M3 when two structures were overlapped. In this situation, determining the exact position was limited and unreliable even for the specialist, as shown in previous studies^25,26^. However, AI could determine both positions more accurately than OMFS specialists.

Until now, if M3 and IAN overlap on panoramic radiograph, specialists could use the known predictive signs of IAN injury to determine the positional relationship whether the two structures were in contact or intimate. Umar et al. compared the positional relationship between IAN and M3 through panoramic radiography and CBCT. Loss of the radiopaque line and diversion of the canal on panoramic radiographs resulted in tooth and nerve contact in 100% of the cases on CBCT. Darkening of the roots were associated with contact on CBCT in 76.9% of the cases studied^27^. However, another study reported that the sensitivities and specificities ranged from 14.6 to 68.3% and from 85.5 to 96.9%, respectively, for those three predictive signs^1^. Datta et al. compared those signs with the clinical findings during surgical removal and found that only 12% of patients with positive radiological signs showed clinical evidence of involvement^3^. In the present study, we adopted CBCT reading results instead of radiological signs on panoramic radiography to determine the positional relationship so that the AI could determine whether the two structures were in contact or intimate, showing an accuracy of 0.55 to 0.72. Compared to another study^1^, our deep learning model exhibited similar performance (accuracy 0.87, precision 0.90, recall, 0.96, F1 score 0.93, and AUC 0.82) to determine whether M3 is contacting the IAN or not. This could explain the different model performance depending on the characteristics of the data.

To replace CBCT with analysis of panoramas with AI, information about bucco-lingual positioning was necessary to ensure safe surgical outcomes. It has been reported that the lingual position of the nerve to the tooth has a significantly higher risk of IAN injury compared to other positions^28^. To the best of our knowledge, no studies have evaluated bucco-lingual positioning through panoramic radiograph because there were no methods to predict this position using one radiograph. Two intraoral radiographs with different angle (vertical tube-shift technique) in the third molar area caused patient discomfort and nausea during placement of the film or sensor of the digital intraoral x-ray devices^29^ and is difficult to use clinically. Since there was no effective method to discern the position, the accuracy of the specialists was low in this study. On the contrary, the AI showed considerably high accuracy ranges from 67.7 to 80.6% despite the small amount of study data. The course of the IAN predominantly is buccal to the tooth^28^, and our data revealed a similar situation. However, the total number of cases was small to match the numbers in each group evenly for deep learning. In addition, the lack of total number of cases forced the use of a simple deep learning model with a relatively small number of parameters to be optimized. Therefore, training AI with more data could produce more accurate results and be used more widely in clinical settings.

In this study, bucco-lingual determination (Experiment 2) exhibited superior performance for true contact positioning (Experiment 1). The difference in accuracy between the two experiments seems to be a characteristic of the data rather than a special technical difference. There might be a particular advantage for AI to be recognized in bucco-lingual classification, or that some of the contact classification data might have characteristics that are difficult to distinguish.

There are several studies that have developed Al algorithms that have been able to outmatch specialists in terms of performance and accuracy. AI assistance improved the performance of radiologists in distinguishing coronavirus disease 2019 from pneumonia of other origins in chest CT^30^. Moreover, the AI system outperformed radiologists in clinically relevant tasks of breast cancer identification on mammography^31^. In the present study, the AI exhibited much higher accuracy and performance compared to those of OMFS specialists. To determine the positional relationship between M3 and IAN, we performed preliminary tests to determine the most suitable AI model using VGG19, DenseNet, EfficientNet, and ResNet-50. ResNet showed higher AUC in Experiment 2 and comparable AUC in Experiment 1 (Supplemental Tables 1–3). Therefore, it was chosen as the final AI model.

This study has limitations. First, the absolute size of the training dataset was small. Data augmentation by image modification was used to overcome the limitation of a small sized dataset. Nevertheless, as shown in Table 1, there were cases where training did not proceed robustly. Therefore, the performances of the trained models highly depend on the train-test split. This unsoundness of the trained model, which hinders the clinical utility of AI models for primary determination in practice, can be alleviated by collecting more data and using them for training. Also, the size of deep learning models is an important factor in performance and, in general, a large number of instances are required to train a large size deep neural network without overfitting. Thus, not only collecting more data but also exploiting external datasets from multiple dental centers can be considered to increase the performance of AI models. However, this study is meaningful in that the AI model performed better than experts even under these adverse conditions. Second, the images used in this study were cropped without any prior domain knowledge such as proper size or resolution to include sufficient information to determine true contact or bucco-lingual positional relationship between M3 and IAN. If the domain knowledge is reflected to construct a dataset, the performances of AI models can be highly increased. Third, the use of interpretable AI models^32^, which can explain the reason for the model prediction, can help to identify the weaknesses of the trained models. The identified weaknesses can be overcome by collecting data that the models have difficulty in classifying. Finally, the various techniques developed in the machine learning society, such as ensemble learning^33^, self-supervised learning^34^, and contrastive learning^35^, can be utilized for further improvement of the performance of our models even in situations where the total number of cases is insufficient as well.

## Conclusions

In this study, we developed and validated a deep learning algorithm that determined positional relationship between M3 and IAN canal at a performance level superior to that of experts. Once tested prospectively in clinical settings, the algorithm could have the potential to narrow patient access to CBCT or prepare for surgical extraction.

## Supplementary Information

Supplementary Tables.

## Data Availability

The data that support the findings this study is available from the authors upon reasonable request.
