# Supplementary material for: Artificial intelligence in positioning between mandibular third molar and inferior alveolar nerve on panoramic radiography
Source: Sci Rep. 2022 Feb 14;12:2456. doi: 10.1038/s41598-022-06483-2 (PMC8844031; doi:10.1038/s41598-022-06483-2)
Supplement: Supplementary file 1 — Supplementary Tables. [file 41598_2022_6483_MOESM1_ESM.docx]

**Supplemental**

**Table 1.** Model performance of five random samplings by VGG19 in each experiment

| **Experiment 1, true contact position between M3 and IAN** | | | | | |
| --- | --- | --- | --- | --- | --- |
| **Work** | **Accuracy** | **Precision** | **Recall** | **F1 score** | **AUC** |
| 1 | 0.64 | 0.59 | 0.47 | 0.52 | 0.71 |
| 2 | 0.54 | 0.62 | 0.28 | 0.39 | 0.61 |
| 3 | 0.58 | 0.58 | 0.29 | 0.39 | 0.70 |
| 4 | 0.62 | 0.58 | 0.50 | 0.54 | 0.69 |
| 5 | 0.68 | 0.67 | 0.40 | 0.50 | 0.70 |
| Average | 0.61 | 0.61 | 0.39 | 0.47 | 0.68 |
| **Experiment 2, bucco-lingual position between M3 and IAN** | | | | | |
| 1 | 0.65 | 0.67 | 0.31 | 0.42 | 0.61 |
| 2 | 0.58 | 0.50 | 0.15 | 0.24 | 0.62 |
| 3 | 0.48 | 0.17 | 0.08 | 0.11 | 0.27 |
| 4 | 0.61 | 0.75 | 0.21 | 0.33 | 0.50 |
| 5 | 0.48 | 0.67 | 0.12 | 0.20 | 0.55 |
| Average | 0.56 | 0.55 | 0.18 | 0.26 | 0.51 |

M3, mandibular third molar; IAN, inferior alveolar nerve; AUC, Area under the ROC curve.

**Table 2.** Model performance of five random samplings by DenseNet in each experiment

| **Experiment 1, true contact position between M3 and IAN** | | | | | |
| --- | --- | --- | --- | --- | --- |
| **Work** | **Accuracy** | **Precision** | **Recall** | **F1 score** | **AUC** |
| 1 | 0.64 | 0.60 | 0.47 | 0.52 | 0.71 |
| 2 | 0.55 | 0.62 | 0.28 | 0.39 | 0.61 |
| 3 | 0.68 | 0.67 | 0.40 | 0.50 | 0.70 |
| 4 | 0.63 | 0.58 | 0.30 | 0.40 | 0.60 |
| 5 | 0.60 | 0.72 | 0.36 | 0.48 | 0.65 |
| Average | 0.62 | 0.64 | 0.36 | 0.46 | 0.65 |
| **Experiment 2, bucco-lingual position between M3 and IAN** | | | | | |
| 1 | 0.42 | 0.00 | 0.00 | 0.00 | 0.67 |
| 2 | 0.48 | 0.00 | 0.00 | 0.00 | 0.70 |
| 3 | 0.55 | 0.33 | 0.08 | 0.13 | 0.54 |
| 4 | 0.61 | 0.67 | 0.29 | 0.40 | 0.64 |
| 5 | 0.48 | 0.67 | 0.12 | 0.20 | 0.60 |
| Average | 0.52 | 0.33 | 0.10 | 0.15 | 0.63 |

M3, mandibular third molar; IAN, inferior alveolar nerve; AUC, Area under the ROC curve.

**Table 3.** Model performance of five random samplings by EfficientNet in each experiment

| **Experiment 1, true contact position between M3 and IAN** | | | | | |
| --- | --- | --- | --- | --- | --- |
| **Work** | **Accuracy** | **Precision** | **Recall** | **F1 score** | **AUC** |
| 1 | 0.63 | 0.61 | 0.36 | 0.45 | 0.71 |
| 2 | 0.63 | 0.73 | 0.42 | 0.53 | 0.60 |
| 3 | 0.56 | 0.47 | 0.76 | 0.58 | 0.62 |
| 4 | 0.67 | 0.66 | 0.54 | 0.59 | 0.70 |
| 5 | 0.52 | 0.60 | 0.21 | 0.31 | 0.61 |
| Average | 0.60 | 0.61 | 0.46 | 0.49 | 0.68 |
| **Experiment 2, bucco-lingual position between M3 and IAN** | | | | | |
| 1 | 0.42 | 0.50 | 0.22 | 0.31 | 0.40 |
| 2 | 0.71 | 0.82 | 0.56 | 0.67 | 0.74 |
| 3 | 0.65 | 0.60 | 0.47 | 0.52 | 0.71 |
| 4 | 0.42 | 0.25 | 0.14 | 0.18 | 0.54 |
| 5 | 0.45 | 0.50 | 0.18 | 0.26 | 0.53 |
| Average | 0.53 | 0.53 | 0.31 | 0.39 | 0.59 |

M3, mandibular third molar; IAN, inferior alveolar nerve; AUC, Area under the ROC curve.
